# Challenges and Potential Solutions of Psychophysiological State Monitoring with Bioradar Technology

**DOI:** 10.3390/diagnostics8040073

**Published:** 2018-10-17

**Authors:** Lesya Anishchenko

**Affiliations:** Remote Sensing Laboratory, Bauman Moscow State Technical University, Moscow 105005, Russia; anishchenko@rslab.ru; Tel.: +7-495-632-2219

**Keywords:** stress detection, bioradar, psychophysiological state monitoring, unobtrusive monitoring

## Abstract

Psychophysiological state monitoring provides a promising way to detect stress and accurately assess wellbeing. The purpose of the present work was to investigate the advantages of utilizing a new unobtrusive multi-transceiver system on the accuracy of remote psychophysiological state monitoring by means of a bioradar technique. The technique was tested in laboratory conditions with the participation of 35 practically healthy volunteers, who were asked to perform arithmetic and physical workload tests imitating different types of stressors. Information about any variation in vital signs, registered by a bioradar with two transceivers, was used to detect mental or physical stress. Processing of the experimental results showed that the designed two-channel bioradar can be used as a simple and relatively easy approach to implement a non-contact method for stress monitoring. However, individual specificity of physiological responses to mental and physical workloads makes the creation of a universal stress-detector classifier that is suitable for people with different levels of stress tolerance a challenging task. For non-athletes, the proposed method allows classification of calm state/mental workload and calm state/physical workload with an accuracy of 89% and 83% , respectively, without the usage of any additional a priori information on the subject.

## 1. Introduction

Stress is a normal organism response to changing environmental conditions, as defined by Selye [1]. In [2], Selye differentiated between “dis- and eustress”, or pathological stress (negative, distress) vs. health-promoting stress (positive, eustress). While eustress helps us deal successfully with everyday challenges, distress leads to physiological and psychological health problems. In the short term, distress may result in fatigue, decrease in the ability to work, anxiety, etc. However, chronic stress, which is one of the fundamental problems of today’s society, may result in irreversible physiological and psychological shifts that increase, in the long-term perspective, the risk of socially-significant health problems such as cardiovascular diseases [3,4], obesity [5], diabetes [6], sleep disorders [7,8], different types of psychosis [9,10] and depression [11]. That is why stress detection techniques may be helpful tools allowing the prevention of health problems associated with prolonged stress. These methods should provide scientifically reliable results as well as be comfortable for the user.

At present, to detect stress and estimate its level, numerous psychological questionnaires are used. The main drawback of their usage is the necessity to interpret results by an expert (professional psychologist). Moreover, there are stress detection techniques based on measuring physiological parameters, such as level of cortisol [12,13], event-related brain potential [14], electrodermal activity or galvanic skin response [15,16], blood pressure [17], heart rate variability (HRV) [18], respiration [19], etc. The main drawback of these methods is their need for direct contact such as electrodes or sensors with the human body or taking saliva samples (in the case of measuring cortisol level), which makes them inappropriate for everyday usage.

Some stress detection methods are based on analyzing information about pupil diameter [20], eye movements [19] and facial expression [21] extracted from the video data. In comparison to contact methods, these are much more comfortable for the user; however, they are known to be extremely sensitive to lighting conditions as all methods are based on analyzing data from video cameras. Therefore, the reliability of their results is questionable.

Furthermore, there are mobile applications and wearables that claim to be able to monitor mental stress [22]. For the majority of them, the main limitation is that they only consider HRV registered by a smartphone camera. Moreover, in the majority of cases, there is no data about the accuracy of HRV and stress detection algorithm verification in realistic conditions for these apps, which reduce the confidence held in the reliability of the results.

One of the methods that can be used for prolonged daily unobtrusive stress detection and monitoring is the bioradar technique [23]. This method has been known since the 1970s [24,25]. It is based on the modulation of a radar probing signal reflected from the human by the movement of a body’s surface, which may be caused by respiration, heartbeat, vocalization, gut motility, limb movements, etc. The main advantage of bioradiolocation is its non-contact nature, since any direct physical contact with the user is not required. Over the last decade, the scientific community and manufacturers have experienced a growing interest in non-contact methods thanks to its high acceptance by patients and users [26].

Research activities dealing with the application of bioradars for the estimation of user’s psychophysiological states and detection of mental stress have been carried out at the Remote Sensing Laboratory of Bauman Moscow State Technical University (BMSTU) since 2011 [27,28].

It should be noted that, until now, the majority of work dealing with the monitoring of a human psychophysiological state by means of bioradars has been mainly focused on bioradar signal processing, which allows the registration of heartbeat and respiration patterns [29,30,31]. In the present paper, we propose using the features of vital signs registered by bioradar for detecting the presence of external stress factors by analyzing them.

This paper deals with two main challenges that arise while applying the proposed technique in realistic conditions and suggests methods for their solution by using the experience of previous works along with new experimental data. The first one is caused by a high impact of the subject orientation toward radar on the level of the desired signal and the accuracy of the estimation of vital signs. The second challenge is determined by the variability of subjects’ reaction to a stressor, which depends on the level of individual stress tolerance.

The purpose of the present study was to investigate the advantages of utilizing a multi-transceiver system on the accuracy of psychophysiological state monitoring by means of a bioradar technique. This is explicitly due to the capability of such bioradar architecture to overcome one of the challenges that arise while applying the proposed technique in realistic conditions, which is a high impact of the subject orientation toward radar antennae on the level of the desired signal and the accuracy of the estimation of vital signs from previous works [32]. The novelty of the present work lies in the proposed architecture of the bioradar using two transceivers, which allows the observation of the subject from different angles, and thus increases classification accuracy compared to using a standard bioradar with a single transceiver. Moreover, the present paper discusses the method to overcome another challenge of using a bioradar for stress detection, which is determined by the variability of subjects’ reaction to a stressor due to individual stress tolerance.

## 2. Materials and Methods

### 2.1. Experimental Setup

**Figure 1:**
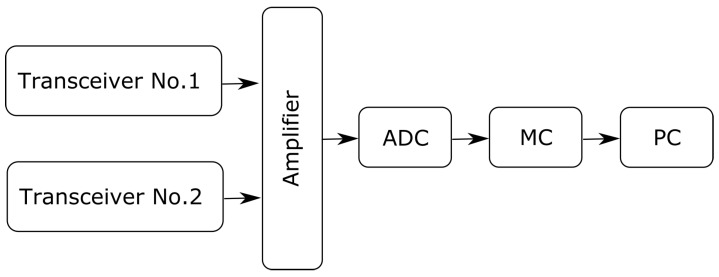
Two-channel bioradar scheme.

The architecture of the bioradar used in the present work is shown in Figure 1.

**Figure 2:**
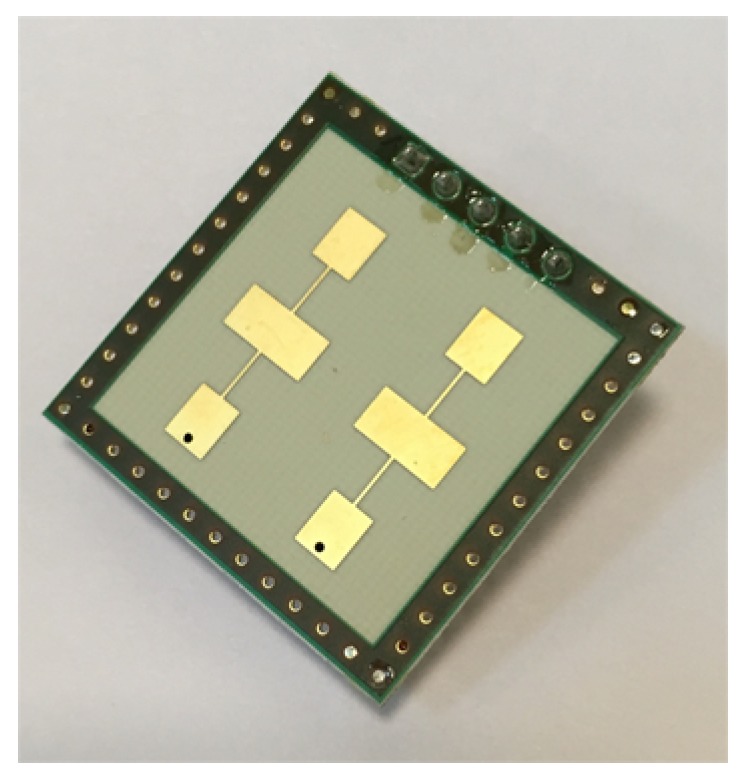
K-LC5 transceiver [33].

The bioradar was designed using two single-chip, high-sensitivity, dual-channel transceivers K-LC5 (RFbeam) [33], a photo of which is shown in Figure 2. The transceivers operate at a frequency of 24 GHz and provide output signals of two quadratures (I and Q). To prevent interference between the probing signals of two transceivers, we use VCO input of the second transceiver to make the probing frequency 24.2 GHz. As the K-LC5 sensors do not have an integrated amplifier, we designed a variable gain amplifier adapted to monitor human vital signs by limiting bandwidth to around 0.1–15 Hz. The gain can be adjusted in the range of 0–30 dB, depending on the range to the examinee and his orientation towards the transceiver antenna.

As an analog-to-digital converter (ADC), we used a higher-precision ADC ADS1115, which provides 16-bit precision at 860 samples/second over I2C and can be configured as four single-ended input channels. As a micro-controller (MC) board, an Arduino UNO board was used, which sent the data registered by the bioradar via Serial Port to the personal computer (PC) for further off-line analysis.

The maximum power density radiated by the radar is less than 3 μW/cm${}^{2}$, which satisfies the Russian standard for microwave emission, which is 25 μW/cm${}^{2}$ in the frequency range 3–300 GHz (for 24 h exposure).

### 2.2. Description of the Experimental Procedure

Experiments were conducted to determine whether the usage of a bioradar with two spaced transceivers increases the accuracy of detecting mental and physical stress in humans as compared to a single transceiver bioradar and to evaluate the corresponding accuracy gain. Using spaced transceivers should allow the observation of a biological object from different angles, which results in different amplitude levels of a received signal. Moreover, such architecture allows separating the vital signs patterns of two different, simultaneously observed humans if needed, which is described in [34].

**Figure 3:**
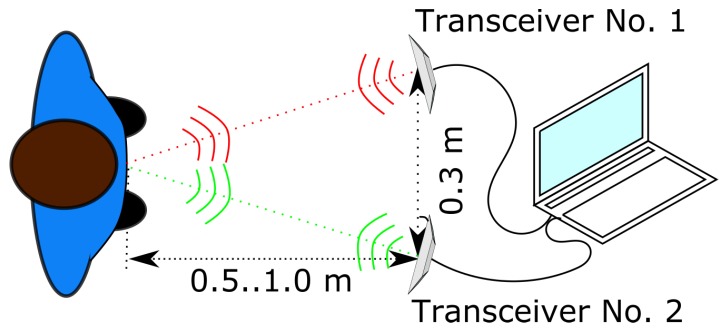
Scheme of the experiment.

During the experiment, a subject sat in front of the bioradar at a distance of 0.5–1.0 m from the antennas. The distance to the subject varied depending on the individual anthropomorphic features of the subject and the way he/she sat in the chair during the experiment. Each transceiver was oriented to the surface of the examinee’s chest, and the distance between centers of the transceiver antennas was 0.3 m. The scheme of the experiment is given in Figure 3.

Experiments were carried out at BMSTU from March to April 2018. For the experiments involving human participants, an ethical approval was obtained on 1 March 2018 from the ethics committee of BMSTU. The test population of 35 young healthy adults consisted of 14 males and 21 females in the age group of 19–22 years. All subjects gave their oral as well as written informed consent prior to the start of the experiments. In addition, each volunteer provided information about his/her individual fitness state, which may affect an organism individual reaction to stress. Four examinees turned out to be athletes.

In the previous work [35], we found that estimates of vital signs made by bioradars are less accurate for overweight people than for people with a normal weight. This is reasonable because the movements of the subjects’ thorax caused by respiration and heartbeat are damped by a subcutaneous fat layer. That is why, in the present work, height and weight measurements were taken for each subject prior to the experiment to calculate their Body Mass Index (BMI). Information about the studied subjects is given in Table 1.

For each volunteer, experiments of three of the following types were carried out.

- Calm breathing test: During this test, an examinee was asked to sit relaxed and breathe normally. If the subject was in a state of psycho-emotional agitation, then vital signs estimation should be performed only after the respiratory and heart rates dropped stationary levels, which corresponded to the calm state of the examinee. It took, in general, between 1 and 2 min for vital signs to stabilize after the beginning of the experiment as shown in [27]. That is why, to prevent the influence of the psycho-emotional agitation of some examinees at the start of the experiment, 2 min were added to the experiment duration. In total, calm breathing test lasted for 5 min; however, only the last 3 min of data were used in further analysis.
- Mental workload test: The volunteer was asked to perform a mental arithmetic task, which was more complex that the one from our previous papers dealing with mental stress monitoring [27,28]. The usage of a more complex arithmetic task was needed to present a challenge that resulted in a physiological response (increasing of cerebral oxygen consumption) in the examinees. The duration of this experimental stage was 3 min for each subject. We did not use standard stress-inducing procedures such as the Trier social stress test because it requires communication with the examinee during the experiments, which may significantly reduce the quality of useful signals registered by the bioradar.
- Exercise tolerance test: Each volunteer was asked to perform some physical exercises (30 bobs or plank exercise for 1 min). After that, the examinee’s vital signs were registered by a bioradar for 3 min.

### 2.3. Signal Processing Technique

The bioradar signal processing algorithm used in the present work was designed utilizing Matlab2018a. It consisted of pre-processing and classification algorithms. The former is required for accurate extraction of the features that are used by the latter for detection of stress.

#### 2.3.1. Pre-Processing Algorithm

**Figure 4:**
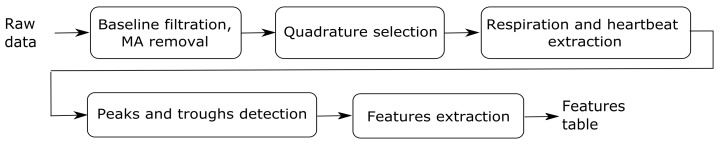
Scheme of the pre-processing algorithm.

The scheme summarizing the steps of the signal pre-processing algorithm is depicted in Figure 4.

**Figure 5:**
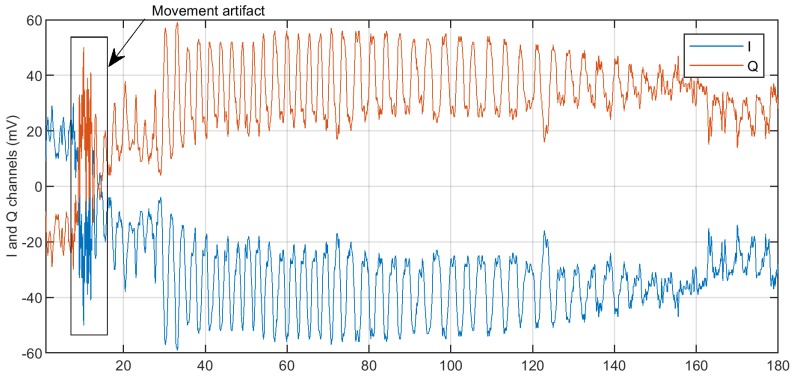
Raw bioradar quadratures.

The first stage consists of the baseline trend and movement algorithm suppression. These tasks are performed utilizing a highpass Butterworth filter with a cut-off frequency of 0.05 Hz for baseline trend filtering, and the algorithm proposed in [36] for movement artifact (MA) removal. In Figure 5, raw quadratures delivered by a single transceiver with the detected movement artifact are shown. After that, the examinee vital signs were registered by a bioradar for 3 min. The artifact periods were excluded from further analysis of the signal.

The second stage deals with the selection quadrature for further analysis over the I and Q channels for two transceivers. As is known, in realistic conditions, phase demodulation of two quadratures received by the radar does not always provide good results due to the clutter caused by reflections from surrounding objects and the walls of the room where the examination takes place. Thus, in the present work, we did not use phase demodulation. Instead, we picked one quadrature with a higher peak-to-peak variation for each transceiver. Selected quadratures were used for further analysis.

After that, respiration and heartbeat patterns were extracted from chosen quadratures by sixth-order bandpass Butterworth filters with bandwidth [0.05; 0.7] Hz and [0.7; 2.0] Hz, respectively.

**Figure 6:**
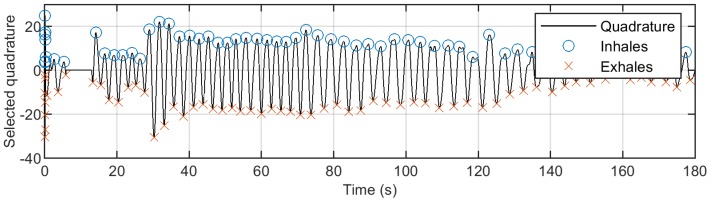
Respiration pattern with detected peaks and troughs.

After filtration, peaks and troughs were detected in extracted respiration and heartbeat patterns by a search of local maximums and minimums using the function findpeak from Signal Processing Toolbox in Matlab. Peaks were detected as turning points in the signal with the minimum distance of 0.5 s and 1.5 s for heartbeat and respiratory patterns, respectively. In Figure 6, the respiration signal filtered from the chosen quadrature is shown. Moreover, the ends of inhaling and exhaling phases, corresponding to the local minimums and maximums of the filtered signal, are depicted.

Time and frequency domain features of respiration and heartbeat patterns filtered from the bioradar signal were extracted for further classification.

**Figure 7:**
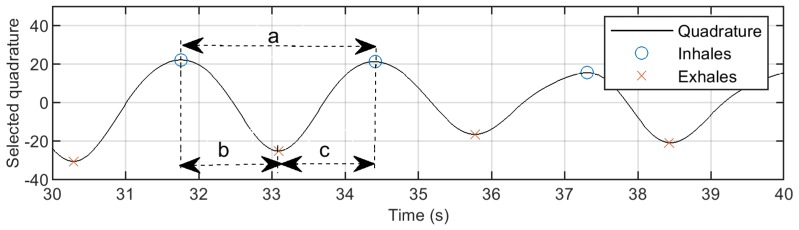
Time features for respiration pattern: respiration circle (a), exhaling (b) and inhaling (c) intervals.

Time Features: The number of positive peaks was computed in a time window of 30 s and 10 s to estimate respiration and heartbeat rates, respectively. For these variables, the average, median, Inter Quartile Range (IQR), median-IQR rate, variance and skewness were computed. In addition, we estimated the same parameters for respiration circles (intervals between peeks), exhaling (time between peaks and troughs) and inhaling (time between troughs and peaks) periods. For more details, see Figure 7.

Frequency Features: The average respiration and heartbeat frequencies were detected by frequency spectrum analysis. A standard Matlab function providing Discrete Fourier Transform was used for this purpose. Estimation of respiratory and heartbeat frequencies was done in a time window of 30 s and 10 s, respectively, by detecting a global maximum in a frequency spectrum in corresponding filtered signals. Such widths of time windows allow considering the analyzed fragments as being quasi-stationary.

#### 2.3.2. Classification Algorithm

To discriminate between the calm breathing test and workload of different types (mental and physical), we used a support vector machine (SVM) classifier with a linear kernel realized in MATLAB. We chose this classifier because it showed the best performance using the cross-validation k-folds technique with k=5 , which was applied to prevent overfitting.

## 3. Results

The classifier was trained to distinguish between the calm state of the examinee and his/her state under mental or physical stress. Firstly, training was carried out using features extracted from data recorded by transceivers No. 1 and 2 (F${}_{tr1}$ , F${}_{tr2}$, respectively) independently. After that, the same was done using a superposition of features for both transceivers (F${}_{tr1\&2}$). To estimate the performance of the proposed classifier, the confusion matrix and accuracy were calculated. The results listed below show how usage of additional transceivers may help to improve the accuracy of examinees psychophysiological state classification.

### 3.1. Classification Calm State/Mental Workload

Table 2 presents the results of classification of steady state/mental stress for using data for a transceiver No. 1. In Table 3, the accuracies of classifiers trained on F${}_{tr1}$, F${}_{tr2}$, and F${}_{tr1\&2}$ are listed. It can be seen that the accuracy of the classifier using features for both transceivers F${}_{tr1\&2}$ is slightly higher than for classifiers using features for a single transceiver.

The classifiers’ relatively low accuracies (less than 80% ) were caused by nine ”outliers”; persons whose reactions to the mental stress was completely different from the other 26 subjects. Their cardiorespiratory system reacted by increasing frequencies of respiration and heartbeat.

Four outliers were experienced swimmers, which is why their cardiorespiratory system responded to the mental workload by increasing amplitudes of respiratory and heart muscles contractions, while the frequencies of these processes remained mostly unchanged, which is typical for trained persons.

Five other outliers had tachypnea. Their respiration rate during the calm breathing test was higher than 0.5 Hz, which is known to be too high for normal calm breathing. These examinees’ respiration and heartbeat systems react to the mental workload by decreasing the analyzed vital signs frequencies. Moreover, eight out of nine outliers had BMI > 25, which may cause less accurate detection of respiration and heartbeat patterns, and thus influence the classifier accuracy.

The re-trained classifier for experimental dataset without nine outliers showed much better performance (Table 4) than the previous one (88.5% for dataset without nine outliers vs. 77.5% for the whole dataset). Moreover, it should be noted that using classification data for both transceivers resulted in higher accuracy than the same estimation using singe transceiver data (88.5% vs. 84.6% ).

### 3.2. Classification Calm State/Physical Workload

Table 5 presents the results of classification of steady state/physical stress using F${}_{tr1}$, F${}_{tr2}$, and F${}_{tr1\&2}$. It can be seen that the accuracy of classifiers using features for both transceivers F${}_{tr1\&2}$ is higher than for classifiers using features for a single transceiver (F${}_{tr1}$ and F${}_{tr2}$).

## 4. Discussion

Psychophysiological state monitoring provides a promising way for detecting stress and accurate assessment of wellbeing. The major advantage of the proposed technique, compared to other stress detection methods, is its unobtrusive nature that does not require any direct contact between the device and the person. The technique was tested in laboratory conditions with the participation of 35 young, healthy volunteers who were asked to perform arithmetic and physical workload tests that imitated different types of stressors. The information about variations of vital signs registered by a bioradar with two transceivers was used to detect mental or physical workload. The usage of two transceivers provides the benefit of observing a subject from different angles, which results in increasing classification accuracy as compared to using a bioradar with a single transceiver. A drawback of the proposed approach might be given by the increasing complexity of the device architecture.

**Figure 8:**
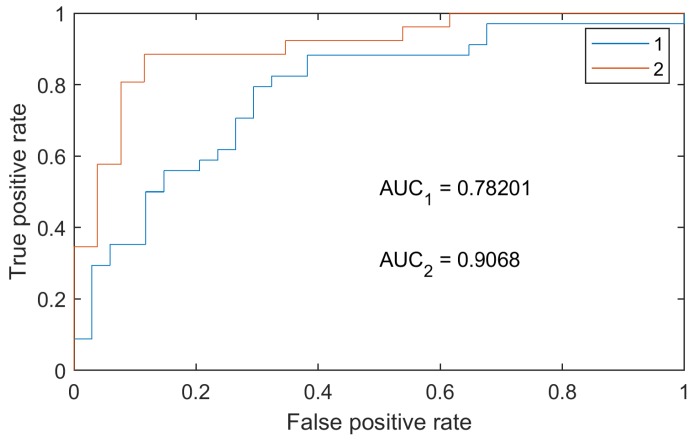
ROC curve of the calm state/mental workload classifier for the whole dataset (1), and for non-athletes dataset (2).

The analysis of the experimental results showed that the physiological responses to mental and physical workload differ for trained and untrained persons as well as for persons with tachypnea. This individual specificity of physiological responses to mental and physical workload makes the creation of a universal stress detector suitable for people with different level of stress tolerance a challenging task. One of the possible solutions of this issue may be training different classifiers for athletes and non-athletes without tachypnea. In the present paper, using such an approach allows increasing accuracy for classification of the calm state/mental workload from 78% to 89% as well as increasing AUC values (Figure 8).

The achieved results should be accepted with caution because the experimental data used for the classifier training are only for young, practically healthy examinees. The relatively low number of volunteers who were declared to be athletes or having tachypnea does not allow the training of the classifier for these groups; however, in the future, we are planning to enrich the experimental dataset and add heuristics to make the classifier consider individual information of the person (BMI, chronic tachypnea, etc.), which should increase the accuracy of psychophysiological monitoring.

The work might contribute to the development of a noncontact system for evaluating individual reactions of a user to mental stress factors in everyday life.

In future work, it is planned to extend the research to the evaluation of different stress levels using standard stress-inducing procedures. This activity will be carried out in cooperation with psychologists and medical researchers from Lomonosov Moscow State University (Moscow, Russia).

## Figures and Tables

**Table 1:** Information about the studied subjects.

Dataset Characteristics	Values
Male : Female	14 : 21
Age (Years)	20.1 ± 0.6 (19–22)
Body Mass Index (kg/m${}^{2}$)	22.0 ± 3.6 (17.4–30.4)
Respiration rate (breath per minute)	16.9 ± 5.0 (7–36)

**Table 2:** Classification results for transceiver No. 1.

		Predicted Class	
		Steady State	Mental Stress
True Class	Steady state	26	9
	Mental stress	9	26
Accuracy, %			
Sensitivity, %		74.3	
Specificity, %			

**Table 3:** Steady state/mental stress classification results.

	F${}_{tr1}$	F${}_{tr2}$	F${}_{tr1\&2}$
Accuracy, %	74.3	64.7	77.5

**Table 4:** Steady state/mental stress classification results (dataset without nine outliers).

	F${}_{tr1}$	F${}_{tr2}$	F${}_{tr1\&2}$
Accuracy, %	84.6	78.8	88.5

**Table 5:** Steady state/physical stress classification results.

	F${}_{tr1}$	F${}_{tr2}$	F${}_{tr1\&2}$
Accuracy (dataset for all 35 examinees), %	69.1	73.5	77.9
Accuracy (dataset without 9 outliers), %	75.0	80.8	82.7

## Abbreviations

The following abbreviations are used in this manuscript:

ADC	Analog-to-Digital Converter
AUC	Area Under the Curve
BMI	Body Mass Index
BMSTU	Bauman Moscow State Technical University
HRV	Heart Rate Variability
I2C	Inter-Integrated Circuit
MA	Movement Artifact
MC	Micro-Controller
PC	Personal Computer
ROC	Receiver Operating Characteristic
SVM	Support Vector Machine
VCO	Voltage-Controlled Oscillator
